# Suicide attempt rate and the risk factors in young, first-episode and drug-naïve Chinese Han patients with major depressive disorder

**DOI:** 10.1186/s12888-022-04254-x

**Published:** 2022-09-16

**Authors:** Gang Ye, Zhe Li, Yan Yue, Yuxuan Wu, Ruchang Yang, Haitao Wang, Siqi Wu, Yue Zhou, Xueli Zhao, Xiaoli Lv, Nian Yuan, Ronghua Li, Guangya Zhang, Pallavi B. Ganapathi, Hanjing Emily Wu, Xiangdong Du, Xiang-Yang Zhang

**Affiliations:** 1 Suzhou Guangji Hospital, The Affiliated Guangji Hospital of Soochow University, No. 11 Guangqian Road, 215137 Suzhou, Jiangsu Province PR China; 2 Medical College of Soochow University, Suzhou, China; 3 School of Psychology and Mental Health, North China University of Science and Technology, Tangshan, China; 4 Xuzhou Medical University, Xuzhou, China; 5 Department of Psychiatry and Behavioral Sciences, The University of Texas Health Science Center at Houston, Houston, TX USA; 6 CAS Key Laboratory of Mental Health, Institute of Psychology, Chinese Academy of Sciences, Beijing, China

**Keywords:** Major depressive disorder, Suicide attempts, Thyroid dysfunction, Metabolic abnormality

## Abstract

**Background:**

In recent years, the rates of suicide among young people have been increasing, and major depressive disorder (MDD) is regarded to be its main cause. Many factors such as thyroid dysfunction and metabolic abnormalities are thought to mediate this process, but the conclusions are inconsistent. This study investigated the rate of suicide attempts and associated risk factors among young, first-episode and drug-naïve Chinese Han patients with MDD.

**Methods:**

A total of 917 patients with MDD (aged 18 ~ 35 years) were recruited. Demographic and clinical data were collected and thyroid function, fasting blood glucose and lipid profiles were measured. The Hamilton Depression Rating Scale-17 items (HAMD-17), Hamilton Anxiety Rating Scale (HAMA), positive symptom subscale of Positive and Negative Syndrome Scale (PANSS) and clinical global impression of severity scale (CGI-S) were adopted to assess depression, anxiety, psychotic symptoms and disease severity respectively.

**Results:**

The rate of suicide attempts was 19.5% in young MDD patients. There were significant differences in age (*p* = 0.003), education level (*p* = 0.001), age of onset (*p* = 0.004) and disease duration (*p* = 0.001) between patients with and without suicide attempts. Compared with patients without suicide attempts, patients with suicide attempts had significantly higher scores on the HAMD-17, HAMA, PANSS positive symptom subscale and CGI-S (all *p* < 0.001). Patients with suicide attempts had significantly higher levels of TSH (*p* < 0.001), TgAb (*p* = 0.004), TPOAb (*p* < 0.001), TG (*p* = 0.016), TC (*p* < 0.001), LDL (*p* < 0.001), and fasting glucose (*p* < 0.001), but significantly lower levels of HDL (*p* < 0.001). Logistic regression analysis showed that marital status (OR = 0.515, 95%CI: 0.280–0.950, *p* = 0.515), disease duration (OR = 1.100, 95%CI: 1.013–1.194, *p* = 0.024), HAMA score (OR = 1.313, 95%CI: 1.205–1.430, *p* < 0.001), CGI-S score (OR = 1.875, 95%CI: 1.339–2.624, *p* < 0.001), levels of FT3(OR = 0.717, 95%CI: 0.536–0.959, *p* = 0.025), TPOAb (OR = 1.004, 95%CI: 1.002–1.006, *p* < 0.001), TC (OR = 1.330, 95%CI: 1.011–1.750, *p* = 0.042) and LDL (OR = 0.736, 95%CI: 0.558–0.971, *p* = 0.030) were all independently associated with suicide attempts in young MDD patients.

**Conclusions:**

In China, the rate of suicide attempts in young patients with MDD is quite high and thyroid dysfunction and metabolic abnormalities may be implicated in its pathogenesis.

## Background

Suicide is an important public health problem worldwide. Globally, lifetime prevalence rates are approximately 9.2% for suicidal ideation and 2.7% for suicide attempt [1]. Although suicide rates are highest in older people across different countries [2], there has been a steady increase in the number of suicides among adolescents and young adults over recent decades [3]. Suicide attempts are more common among young people than the elderly, whereas completed suicide is more common among the elderly [4].In many countries, the incidence of suicide attempts is highest in individuals 18–34 years of age [5]. In China, in addition to the peak of over-65 age group, it also has a second smaller peak in the individuals 15 to 34 years of age [6]. Thus, there is an urgent need for us to better understand the characteristics and risk factors of suicide in young adults. However, as far as we know, there is a lack of research in this field.

It is often stated that over 90% of people who die by suicide have mental disorders, particularly major depressive disorder (MDD) [7]. Suicidality is common and regarded as the most serious symptom of MDD. A meta-analysis found that the lifetime prevalence of suicide attempts was 31% and one year prevalence was 8% in MDD patients [7]. The prevalence of suicide attempts in adolescents and young adult MDD patients was from 31.5% to 43.7% in different studies [8, 9]. However, in China, the suicide attempt rate of young adults with MDD is still not clear.

The causes of suicide attempt are complex, relating to numerous biological, clinical, psychological and environmental variables. Previous studies have discussed the associations between thyroid hormones or metabolism biomarkers and suicide attempts [10]. Of these, most supported that thyroid dysfunction and metabolic abnormality played an important role in suicide. At the same time, a series of existing studies reported that patients with MDD had a higher prevalence of thyroid dysfunction [11] and metabolic syndrome than the general population [12]. These findings indicate high pathophysiological overlap between the two conditions. However, the results from different studies are not consistent, and there is a lack of research on young adult patients with MDD, exploring the underlying factors leading to suicide attempts.

Therefore, the aim of this cross-sectional study is to explore the rate and related risk factors, including thyroid hormones levels and metabolic biomarkers, of suicide attempts in young, first-episode and drug-naïve Chinese Han patients with MDD.

## Participants and methods

### Participants

This study was conducted in the psychiatric outpatient department of a general hospital in Taiyuan, Shanxi Province, China during 2015–2017. We approached all outpatients and then collected all cases that met the inclusion and exclusion criteria consecutively, so the sample was randomized. A total of 917 young adult patients with MDD (male/female = 351/566) were recruited. The following inclusion criteria were applied: (1) aged 18 ~ 35 years, Han Chinese; (2) diagnosed with MDD according to the Diagnostic and Statistical Manual of Mental Disorders, Fourth Edition (DSM-IV); (3) this being the first episode; (4) no history of treatment by any antidepressant; (5) the ability to participate in the clinical assessment.

All subjects were in good physical health. Participants were excluded if they had any intake of medication that could affect thyroid function, blood glucose or blood lipid levels. Other exclusion criteria included comorbid psychiatric disorders, drug or alcohol abuse, neurodegenerative and neurological disorders, and pregnant or breastfeeding women. Patients who could not sign informed consent were also excluded.

The sample size was calculated using the formula, *n* = Z^2^p(1-p)/d^2^. *n* = number of sample size; Z = 95% confidence interval equal to 1.96; d = 0.05 (5%), marginal error; *p* = expected prevalence, equal to 0.315 according to a previous Chinese study [8]. This gave an estimate for a sample of 331 patients. In our study, a total of 995 young patients were screened and only those who met the inclusion and exclusion criteria were recruited. Finally, 917 subjects participated in this study. The other 78 participants were excluded because they: (1) were unable to provide written informed consent (*n* = 21); (2) were pregnant or lactating (*n* = 10); (3) had severe physical diseases (*n* = 9); (4) had a substance abuse disorder or dependence (*n* = 9); (5) had a severe personality disorder (*n* = 15); (6) were unable to be interviewed due to an acute clinical condition (*n* = 5); and (7) other unknown reasons (*n* = 9). The sample size for this study was 917, which was markedly larger than the required sample size (*n* = 331), suggesting that our sample size had sufficient power.

In general, the age range of young adults in different countries and organizations ranges from 15–45 years old, with the National Bureau of Statistics of China defining 15–34 years old as young adults. This study was conducted in the context of Chinese culture, with reference ring to the age criteria defined by the National Bureau of Statistics of China. Children and adolescents were not recruited in this study, so we defined 18–35 years old as young adults. More importantly, many studies also defined 18–35 years as young adults [13–15].

This study was approved by the Institutional Review Board of the First Clinical College of Shanxi Medical University (No. 2016-Y27). Following a complete description of the study protocol and procedures to each participant by a psychiatrist or research coordinator, then written informed consent was obtained.

### Clinical measurements

The demographic and clinical data were collected from each subject using a detailed questionnaire. In this study, suicide attempt was defined as self-injurious behavior associated with an intention to end one’s own life but not leading to death. All subjects and/or their family members were asked “In your (or the patient’s) lifetime, did you (he or she) ever attempt suicide?” If they answered “yes”, these patients were regarded as suicide attempters. For patients with suicide attempts, further details were collected, including: the number of suicide attempts, the exact date and the method by which suicide was attempted.

The Hamilton Depression Rating Scale (HAMD-17) and Hamilton Anxiety Rating Scale (HAMA) were utilized to assess depression and anxiety symptoms respectively. The positive symptom subscale of Positive and Negative Syndrome Scale (PANSS) was used to assess psychotic symptoms. The clinical global impression of severity scale (CGI-S) was used to assess disease severity.

The two psychiatrists who participated in this study were trained in the assessment of the HAMD-17 scale, the HAMA-14 scale, and the PANSS positive subscale prior to clinical assessment. After training, the intraclass correlation (ICC) in the two psychiatrists for clinical assessments of three clinical assessment scales all exceeded 0.8.

### Biomarker measurements

The biochemical markers were tested at admission before patients received any treatment. Blood samples were collected in the morning after an overnight fast on the day of clinical data collection. All samples were sent to the laboratory center of the hospital immediately and measured on the same day. Thyroid stimulating hormone (TSH), free triiodothyronine (FT3), free thyroxine (FT4), antithyroglobulinand (TgAb), thyroid peroxidases antibody (TPOAb), triglycerides (TG), total cholesterol (TC), low-density lipoprotein (LDL), high-density lipoprotein (HDL), and fasting blood glucose were measured.

### Statistical analysis

Data analysis was performed using the Statistical Package for the Social Sciences (SPSS version 22). The rate of suicide attempt was expressed as a proportion (percentage). Based on whether patients had past suicide attempts, all subjects were divided into those with suicide attempts (SA) and those without suicide attempts (NSA) MDD groups. Differences in sociodemographic, clinical and biomarker variables between the two groups were assessed by independent t-tests for continuous measures, and by Chi-square tests for categorical variables. Binary logistic regression was conducted to examine which factors had significant influence on suicide attempts in young adult patients with MDD. All comparisons between the two groups were two-sided with a significance level of 5%.

## Results

### Suicide attempts rate and demographic, clinical characteristics

Among the 917 young adult patients with MDD, 179 had suicide attempts. The rate of suicide attempts in this study was 19.5%. We found that 79.3%, 16.8%, and 3.9% of suicide attempt subjects had committed suicide once, twice, and three or more times, respectively. 36.9% of these subjects attempted to kill themselves by cutting wrist, 30.7% by drug overdose, 12.8% by jumping. 65.9% of the subjects attempted suicide within 2 weeks. Table 1 shows the demographic and clinical characteristics of the SA and NSA MDD groups. There were significant differences in age (*p* = 0.003), education level (*p* = 0.001), age of onset (*p* = 0.004) and disease duration (*p* = 0.001) between the two groups. The SA group was older and the age of onset was later. Compared with NSA MDD group, these patients also had longer disease duration. The scores of HAMD-17, HAMA, PANSS positive symptom subscale and CGI-S were significantly higher in SA MDD group than those in NSA MDD group (all *p* < 0.001).

**Table 1:** Demographic and clinical variables in patients with and without suicide attempts

	**NSA MDD group** **(*n* = 738)**	**SA MDD group** **(*n* = 179)**	***t* or *χ2***	***P***
Age (years, Mean ± SD)	24.42 ± 5.39	25.75 ± 5.48	-2.95	0.003
Gender – male, n (%)	287(38.9)	64(35.8)	0.60	0.439
Education level				
Junior high school or below, n (%)	36(4.9)	21(11.7)	15.56	0.001
Senior high school, n (%)	366(49.6)	73(40.8)		
University degree, n (%)	280(37.9)	65(36.3)		
Postgraduate degree, n (%)	56(7.6)	20(11.2)		
Marital status				
Single, n (%)	391(53)	90(50.3)	0.42	0.516
Married, n (%)	347(47)	89(49.7)		
BMI(kg/m^2^, Mean ± SD)	24.31 ± 1.86	24.28 ± 2.61	0.16	0.873
Age of onset (years, Mean ± SD)	24.34 ± 5.35	25.62 ± 5.44	-2.86	0.004
Disease duration (months, Mean ± SD)	4.76 ± 3.11	5.83 ± 4.05	-3.31	0.001
HAMD-17( Mean ± SD)	29.66 ± 2.78	32.23 ± 2.77	-11.10	< 0.001
HAMA( Mean ± SD)	20.02 ± 3.04	23.37 ± 3.45	-12.86	< 0.001
PANSS positive symptom subscale ( Mean ± SD)	8.19 ± 3.40	10.92 ± 6.17	-5.70	< 0.001
CGI-S ( Mean ± SD)	5.8 ± 0.71	6.44 ± 0.73	-10.74	< 0.001

*Note*: *NSA MDD* Major depressive disorder without suicide attempts, *SA MDD* Major depressive disorder with suicide attempts, *BMI* Body mass index, *HAMD-17* Hamilton depression rating scale -17 items, *HAMA* Hamilton anxiety rating scale, *PANSS* Positive and negative syndrome scale, *CGI-S* Clinical global impression of severity scale

### Biochemical parameters of SA MDD group vs. NSA MDD group

Table 2 shows the characteristics of thyroid hormones levels and metabolism biomarkers of the SA and NSA MDD groups. The levels of TSH (*p* < 0.001), TgAb (*p* = 0.004), TPOAb (*p* < 0.001), TG (*p* = 0.016), TC (*p* < 0.001), LDL (*p* < 0.001) and fasting blood glucose (*p* < 0.001) were significantly higher in SA MDD group than those in NSA MDD group, while the levels of HDL (*p* < 0.001) were significantly lower in SA MDD group than those in NSA MDD group. After comparing the biological indicators of the subjects who attempted suicide within two weeks versus two weeks ago, we found that there were no significant differences in metabolic and thyroid function indicators between the two groups except TPOAb (117.15 ± 245.47 vs 200.74 ± 247.14 IU/L, *p* = 0.034).

**Table 2:** Biochemical parameters of SA MDD group vs. NSA MDD group

	**NSA MDD group** **(*n* = 738)**	**SA MDD group** **(*n* = 179)**	***t***	***P***
TSH(uIU/mL, Mean ± SD)	4.46 ± 2.27	6.52 ± 2.90	-8.84	< 0.001
FT3(pmol/L, Mean ± SD)	4.96 ± 0.72	4.85 ± 0.78	1.77	0.076
FT4(pmol/L, Mean ± SD)	16.75 ± 3.04	16.85 ± 3.26	-0.38	0.704
TgAb(IU/L, Mean ± SD)	72.72 ± 198.38	142.93 ± 307.92	-2.91	0.004
TPOAb(IU/L, Mean ± SD)	46.31 ± 94.27	145.64 ± 248.54	-5.26	< 0.001
TG(mmol/L, Mean ± SD)	2.09 ± 0.97	2.29 ± 1.05	-2.41	0.016
TC(mmol/L, Mean ± SD)	5.01 ± 1.06	5.79 ± 1.11	-8.72	< 0.001
LDL(mmol/L, Mean ± SD)	2.87 ± 0.85	3.17 ± 0.97	-4.13	< 0.001
HDL(mmol/L, Mean ± SD)	1.25 ± 0.27	1.13 ± 0.29	4.97	< 0.001
Fasting blood glucose (mmol/L, Mean ± SD)	5.32 ± 0.61	5.57 ± 0.72	-4.18	< 0.001

*Note*: *NSA MDD* Major depressive disorder without suicide attempts, *SA MDD* Major depressive disorder with suicide attempts, *TSH* Thyroid stimulating hormone, *FT3* Free triiodothyronine, *FT4* Free thyroxine, *TgAb* Anti-thyroglobulinand, *TPOAb* Thyroid peroxidases antibody, *TG* Triglyceride, *TC* Total cholesterol, *LDL* Low-density lipoprotein cholesterol, *HDL* High-density lipoprotein cholesterol

### The risk factors for suicide attempts in young adult patients with MDD

We included all variables in the regression analysis to identify predictors of suicide attempts. As shown in Table 3, marital status (OR = 0.515, 95%CI: 0.280–0.950, *p* = 0.515), duration of disease (OR = 1.100, 95%CI: 1.013–1.194, *p* = 0.024), HAMA score (OR = 1.313, 95%CI: 1.205–1.430, *p* < 0.001), CGI-S score (OR = 1.875, 95%CI: 1.339–2.624, *p* < 0.001), levels of FT3 (OR = 0.717, 95%CI: 0.536–0.959, *p* = 0.025), TPOAb (OR = 1.004, 95%CI: 1.002–1.006, *p* < 0.001), TC (OR = 1.330, 95%CI: 1.011–1.750, *p* = 0.042) and LDL (OR = 0.736, 95%CI: 0.558–0.971, *p* = 0.030) were all independently associated with suicide attempts in young MDD patients.

**Table 3:** Risk factors for suicide attempts in young adult patients with MDD

Variable	B	S.E	Wald	*P*	OR (95%CI)
Age	-0.119	0.399	0.089	0.765	0.887(0.406–1.942)
Gender	-0.093	0.211	0.194	0.660	0.911(0.603–1.378)
Education level	-0.049	0.139	0.125	0.723	0.952(0.726–1.249)
Marital status	-0.663	0.312	4.518	0.034	0.515(0.280–0.950)
BMI	-0.084	0.050	2.794	0.095	0.919(0.833–1.015)
Age of onset	0.207	0.400	0.266	0.606	1.229(0.561–2.694)
Disease duration	0.095	0.042	5.096	0.024	1.100(1.013–1.194)
HAMD-17	0.036	0.056	0.414	0.520	1.037(0.929–1.158)
HAMA	0.272	0.044	38.831	< 0.001	1.313(1.205–1.430)
PANSS positive symptom	-0.054	0.028	3.811	0.051	0.947(0.897–1.000)
CGI-S	0.628	0.172	13.425	< 0.001	1.875(1.339–2.624)
TSH	0.085	0.057	2.247	0.134	1.089(0.974–1.217)
FT3	-0.332	0.148	5.018	0.025	0.717(0.536–0.959)
FT4	0.009	0.033	0.079	0.779	1.009(0.946–1.077)
TgAb	-0.001	0.001	2.385	0.123	0.999(0.998–1.000)
TPOAb	0.004	0.001	24.379	< 0.001	1.004(1.002–1.006)
TG	-0.062	0.112	0.309	0.578	0.939(0.754–1.171)
TC	0.285	0.140	4.147	0.042	1.330(1.011–1.750)
LDL	-0.306	0.141	4.690	0.030	0.736(0.558–0.971)
HDL	-0.625	0.378	2.730	0.098	0.535(0.255–1.123)
Fasting blood glucose	0.047	0.176	0.070	0.791	1.048(0.742–1.478)

*Note*: *BMI* Body mass index, *HAMD-17* Hamilton depression rating scale -17 items, *HAMA* Hamilton anxiety rating scale, *PANSS* Positive and negative syndrome scale, *CGI-S* Clinical global impression of severity scale, *TSH* Thyroid stimulating hormone, *FT3* Free triiodothyronine, *FT4* Free thyroxine, *TgAb* Anti-thyroglobulinand, *TPOAb* Thyroid peroxidases antibody, *TG* Triglyceride, *TC* Total cholesterol, *LDL* Low-density lipoprotein cholesterol, *HDL* High-density lipoprotein cholesterol

## Discussion

To our knowledge, this is the first study to explore the association between thyroid hormones levels, metabolism indicators and suicide attempts in young (aged 18 ~ 35 years), first-episode and drug-naïve Chinese Han MDD patients with a large-scale cross-sectional design. Our study found: (1) the rate of suicide attempts in young Chinese MDD patients was 19.5%; (2) Compared with patients without suicide attempts, patients with suicide attempts had older age, later onset age, longer disease duration, higher scores of HAMD-17, HAMA, PANSS positive symptom subscale, CGI-S, higher levels of TSH, TgAb, TPOAb, TG, TC, LDL, fasting blood glucose and lower levels of HDL; (3) The significant associated predictors of suicide attempts were marital status, disease duration, score of HAMA, score of CGI-S, levels of FT3, TPOAb, TC and LDL.

Despite a one-third reduction in the global suicide rate in recent years, the rates of suicide in young people are increasing with a lifetime prevalence of suicidal ideation of 12.1–33%, and of suicidal behavior of 4.1–9.3% [5, 16]. As we know, there is a close correlation between suicidality and depression in the general population. Between 20 and 65% of patients with MDD who died by committing suicide have had a history of previous suicide attempts [17]. The most probable period for the onset of the first episode of MDD extends from mid-adolescence to mid-40 s, but almost 40% experience their first episode of depression before age 20 years, with an average age of onset in the mid-20 s (median 25 years) [18]. This suggests that we need to pay close attention to the correlation between depression and suicide in young people, exploring relevant risk factors, and promoting the formulation of prevention and intervention measures.

In our study, the rate of suicide attempts in young patients with MDD was 19.5% which was similar to that in common MDD population. For example, Ruengorn et al. found that 16.9% patients with MDD attempted suicide [17]; Kim et al. showed that 19.8% patients had a history of at least 1 suicide attempt [19]. A meta-analysis based on Chinese population indicated that the lifetime prevalence of suicide attempts was 23.7% and one-month prevalence was 20.3% [20]. However, Lalthankimi et al. reported the suicide attempts rate in patients with MDD was 35% in their investigation [21], which was higher than that found in our study. Worse, Goodwin et al. found around 15–20% of depressive patients ended their lives by suicide [22]. Only a few studies focused on the incidence of suicide attempts in young patients with depression. A study based on the Korean population (the CRESCEND study) showed that 4.9% young patients had a suicide attempt during their current depressive episode and 43.7% had a history of suicide attempts [9]. This was not consistent with the results of our study. Several factors might contribute to this difference in suicide attempt rates, particularly different sources of subjects. In our study, we only included first-episode and drug-naïve MDD patients while the CRESCEND study included patients with MDD, regardless of whether the depressive symptoms were first-onset or recurrent. Other factors such as genetic and cultural variations, as well as the availability of high-lethality suicide methods and the use of different suicide assessment methods may also add to this variation.

In this study, we found young MDD patients with suicide attempts had older age, later age of onset, and longer duration of disease; these results were not completely consistent with those of some previous studies. These prior studies reported that younger age [4] and earlier age of onset [23] were associated with increased suicide attempts of MDD patients. These differences might be due to that we only focused on young adult patients while the other studies included general population. According to the previous studies [4], within the general population, suicide attempts were more common in the younger age groups. Thus, when compared to patients in the full range of age groups within a population, these patients appear to be younger and have an earlier age of onset. Our study suggested that even within the group of young patients, age and onset age still had impacts on suicide risk. Similar to other studies [24, 25], young MDD patients with suicide attempts had longer disease duration. Our study found there were significant differences in some clinical features between the young patients with and without suicide attempts. Overall, young patients with suicide attempts had higher levels of depression and anxiety and were more likely to have psychotic symptoms. Previous studies agreed that high, severe depression was associated with suicide attempts of patients [4, 21, 26, 27]. However, it remains controversial whether or not comorbid anxiety disorders increase risk of a suicide attempt. A systematic review demonstrated that comorbid anxiety disorder was associated with suicide [28]. Some studies also found the suicide attempt rate of MDD patients with anxiety symptoms was higher than that in those without anxiety symptoms [24, 26]. Yet, some studies have shown no such link between anxiety symptoms and suicide attempts, with several even reporting that anxiety symptoms are protective factors against suicidal behavior in MDD patients [29]. For some patients with depression, anxiety, particularly the agitation that may accompany it, increases the severity of the disease, which in turn increases the risk of suicide. The correlation between anxiety and suicide can also be explained by the presence of substance abuse in patients with high anxiety levels [29]. It should be noted that anxiety may be protective if it associated with fear of death or illnesses [29]. Psychotic symptoms also increase the severity of depression, and some patients may commit suicide under the control of hallucinations and delusions. A recent systematic review and meta-analysis indicated psychotic MDD patients were twice as likely to attempt suicide during both the acute phase and throughout their lifetimes as were MDD patients without psychotic symptoms [30].

The exact mechanism underlying suicidality in MDD remains unknown. A number of studies have explored various biological factors and tried to narrow down certain biomarkers to predict suicide in MDD patients. So far, there is no consistent conclusion. The roles of thyroid dysfunction both in depression and suicide have long been discussed. People with thyroid diseases are more likely to have depressive symptoms, and depression can be accompanied by subtle thyroid abnormalities [31]. Moreover, a recent systematic review and meta-analysis demonstrated patients with suicidal behavior had significantly lower mean FT3 and TT4 levels when compared to patients without suicidal behavior [32]. Compared with patients without suicide attempts, we found patients with suicide attempts had higher levels of TSH, TgAb and TPOAb, which was in agreement with some previous studies. Shen et al. indicated that patients with suicide attempts had higher serum levels of TSH, TgAb and TPOAb [27]. Inconsistent with other studies [33, 34], we did not find significant differences in T3 or T4 levels between the patients with and those without suicide attempts. This implied that subclinical hypothyroidism seemed to play an important role in suicidality of MDD patients. Our findings echo those of Lang et al., who found that subclinical hypothyroidism was related to attempted suicide in MDD patients [11]. We hypothesize that the slowing of thyroid function is a gradual process. This process may start with the increase in TSH and progressively affect T3 and T4 levels due to the compensatory function of the thyroid. For young patients, this change in thyroid function may still be in the early stages, so they mainly show abnormal TSH levels. TPOAb and TgAb are produced due to the damage of thyroid cells and the overflow of "peroxidase (key enzyme for synthesizing thyroid hormone)" and "thyroglobulin" in the cytoplasm into the blood to stimulate the body. They are marker antibodies of autoimmune thyroiditis. The increase of their levels indicates that the thyroid tissue is in an active state of immune inflammation. A large number of studies have found that dysregulation of immune system is involved in the pathophysiology of not only depression [35–37], but also suicide [38–40]. Previously, the occurrence of suicide was associated with autoimmune diseases, including thyroid-specific diseases [32]. According to previous studies, suicide may be more closely associated with autoimmune hypothyroidism rather than autoimmune hyperthyroidism [32]. Patients with a history of suicide attempts showed reduced basal FT4 levels and FT4/FT3 ratio [33]. In addition, there is evidence that thyroid hormones may play a role in the regulation of neurotransmitters (e.g., 5-HT and NE) that are involved in the pathogenesis of suicide [32]. Thyroid hormones may play a compensatory role in the early stages to prevent further development of depression. However, in a long-term depression, the thyroid gland may lose its compensatory role, leading to hypothyroidism [41]. More importantly, previous studies have found that TSH, TGAB and TPOAB may also increase the risk of suicide by exacerbating anxiety, depressive and psychotic symptoms [27].

Another important finding of our study was that patients with suicide attempts had higher levels of TG, TC, LDL, fasting blood glucose and lower levels of HDL. In fact, TC and LDL may be potential predictors of suicide attempts of MDD patients. This was consistent with the findings of several previous studies. A series of studies have found metabolic syndrome is not only correlated with depression, but also associated with increased suicide risk [42]. The associations between lipid profile and suicide attempts in MDD patients have already been widely investigated but the conclusions are not consistent. For example, Fiedorowicz et al. indicated that high cholesterol level was associated with increased risk of suicide attempts in patients with depression [43]. Messaoud et al. found that there was a significant decrease in plasma cholesterol levels in suicidal depressive patients, but the other lipid levels didn’t show any significant differences [44]. da Graça et al. didn’t find any significant difference in TC, HDL or LDL levels between mood disorder (including MDD) patients with and without suicide attempts [45]. Koponen et al. found total and LDL cholesterol and TG levels were higher in depression patients with suicidal behavior [46]. Conversely, Baek et al. found low serum TG and high HDL levels were associated with recent suicide attempt or recent suicide status in MDD patients [47]. A patient’s lipid profile can be influenced by many factors, including age, gender, disease duration, comorbidity, drug use, diet and lifestyle choices. We recruited first-episode and drug-naïve young MDD patients to avoid the influence of many confounding factors. This may partially explain the differences between our findings and other studies. Another possible reason for the inconsistency is that lipid profile may have bidirectional effects on suicide. This has already been proven by some studies. For example, one cohort study done in Taiwan reported a J-shaped association between HDL and suicide risk; both low and high levels of HDL were associated with suicide [48].The biological mechanisms underlying the association between lipid profile and suicide in MDD patient are still not clear. The possible effects of lipid profile on the serotonin system may play an important role [47]. Zhang et al. reported that sequence variants in the serotonin receptor (HTR5A) were associated with a high plasma TG level, meaning a potential brain-specific regulation of plasma TG levels, possibly by alteration of the expression of HTR5A [49]. Papakostas et al. proposed that changes in cholesterol in both directions may lead to alterations in serotonergic function [50]. Comings et al. found a link between serum cholesterol levels and a polymorphism in the promoter region of the serotonin transporter gene [51]. Some studies have found that serum cholesterol levels may be associated with serotonergic receptor function through cortisol and prolactin responses to meta-chlorophenylpiperazine (mCPP) neuroendocrine challenges [52], while others have found that serum cholesterol levels and suicidal behavior are associated with IL-2, which may inhibit melatonin secretion from the pineal gland [53]. In our study, the level of fasting blood glucose was higher in the MDD patients with suicide attempts, which was consistent with some previous studies. The above cited study by Koponen et al. reported that glucose levels correlated positively with the prevalence of suicide [46]. A possible pathophysiological mechanism for the emergence of abnormal glucose metabolism in depression and suicide might lie in the interaction between serotonin and proinflammatory cytokines [46]. The association found between blood glucose levels and suicide may be related to a cytokine-mediated inflammatory process that results in the activation of indoleamine 2,3-dioxygenase (IDO), the depletion of tryptophan and suicidal behavior associated with emerging serotonergic hypofunction, depression and impulsivity [46]. More importantly, we found that in general, there were no significant differences between recent and previous suicide attempt groups in terms of thyroid hormone levels and metabolic biomarkers in this study, suggesting that thyroid function and metabolic indicators may be trait indicators rather than state indicators of suicide in patients with MDD.

There are several limitations in the present study. Firstly, due to the cross-sectional study design, it is not clear whether there is a causative association between suicide attempts and these risk factors. Secondly, we collected information about suicide mainly through interviews with patients and/or their family members, rather than using structured suicide assessment tools. On one hand, this way could be affected by recall bias; on the other hand, it was impossible to evaluate suicide quantitatively. Thirdly, many suicide risk factors found in previous studies were not included in this study, making it difficult to rule out the influence of some confounding factors. Fourthly, a broad range of factors stand to affect the levels of thyroid hormone and metabolic indicators, and their levels might change over time. There was a lack of dynamic assessment in our study. Finally, the participants in our study were from an outpatient psychiatric department of a general hospital. Therefore, the results of this study cannot be generalized to other hospitals or regions.

## Conclusions

In conclusion, our study found that the rate of suicide attempts in young (aged 18 ~ 35 years), first-episode and drug-naïve Chinese Han patients with MDD was 19.5%, suggesting the importance of regular assessment and early intervention of suicide. Moreover, we found some biological, clinical, psychological factors which may be associated with suicide attempts in young MDD patients, including older age, later age of onset, longer disease duration, severity of illness, anxiety symptoms, psychotic symptoms, levels of TSH, TgAb, TPOAb, TG, TC, LDL, HDL and fasting blood glucose. Marital status, disease duration, anxiety symptom, severity of illness, levels of FT3, TPOAb, TC and LDL might be potential predictors of suicide attempts of MDD patients. These findings may provide effective targets for suicide intervention in young MDD patients. Owing to the study constraints mentioned above, our findings should be interpreted within the context of these limitations. Therefore, future investigations are warranted to confirm the present findings using a longitudinal design.

## Data Availability

The data that support the findings of this study are available on request from the corresponding author upon reasonable request.

## Abbreviations

BMI: Body mass index
CGI-S: Clinical global impression of severity scale
DSM-IV: Diagnostic and Statistical Manual of Mental Disorders, Fourth Edition
FT3: Free triiodothyronine
FT4: Free thyroxine
LDL: Low-density lipoprotein cholesterol
HAMA: Hamilton anxiety rating scale
HAMD-17: Hamilton depression rating scale -17 items
MDD: Major Depressive Disorder
HDL: High-density lipoprotein cholesterol
NSA MDD: Major depressive disorder without suicide attempts
PANSS: Positive and negative syndrome scale
SA MDD: Major depressive disorder with suicide attempts
SPSS: Statistical Package for the Social Sciences
TC: Total cholesterol
TG: Triglyceride
TgAb: Anti-thyroglobulinand
TPOAb: Thyroid peroxidases antibody
TSH: Thyroid stimulating hormone
